# Occupational burnout in Iranian health care workers during the COVID-19 pandemic

**DOI:** 10.1186/s12888-022-04014-x

**Published:** 2022-05-28

**Authors:** Mahsa Kamali, Marzieh Azizi, Mahmood Moosazadeh, Hossein Mehravaran, Roya Ghasemian, Maryam Hasannezhad Reskati, Forouzan Elyasi

**Affiliations:** 1 Pediatric Infectious Diseases Research Center, Communicable Diseases Institute, Mazandaran University of Medical Sciences, Sari, Iran; 2 Department of Reproductive Health and Midwifery, School of Nursing and Midwifery, Tehran university of Medical Sciences, Tehran, Iran; 3 Sexual and Reproductive Health Research Center, Mazandaran University of Medical Sciences, Sari, Iran; 4 Gastrointestinal Cancer Research Center, Non-communicable Diseases Institute, Mazandaran University of Medical Sciences, Sari, Iran; 5 Department of Internal Medicine, Pulmonary and Critical Care Division, Faculty of Medicine, Mazandaran University of Medical Sciences, Sari, Iran; 6 Antimicrobial Resistance Research Center, Department of Infectious Diseases, Mazandaran University of Medical Sciences, Sari, Iran; 7 Educational Psychology, Research Ethics Committee, Imam khomeini Hospital, Mazandaran University of Medical Sciences, Sari, Iran; 8 Sexual and Reproductive Health Research Center, Addiction Institute, Mazandaran University of Medical Sciences, Sari, Iran; 9 Department of Psychiatry, Faculty of Medicine, Mazandaran University of Medical Sciences, Sari, Iran; 10 Psychosomatic Ward, Imam Khomeini General Hospital, Razi Ave, Sari, Mazandaran Iran

**Keywords:** Mental health, Occupational health, COVID-19, Burnout

## Abstract

**Background and aim:**

Health care workers (HCWs), mostly frontliners, are encountering numerous physical and psychosocial stressors, and even managing some conflicts over the course of the novel coronavirus disease 2019 (COVID-19). In this respect, the present study was to investigate the prevalence rate of occupational burnout (OB) in such workers during this pandemic.

**Materials and methods:**

This cross-sectional study was conducted between April 6 and May 30, 2020, via an online survey in 31 provinces of Iran, on HCWs selected based on convenience sampling method. For data collection, a socio-demographic information form and the Maslach Burnout Inventory (MBI) was utilized. Descriptive statistics, Chi-square test, and multivariate regression analysis were also applied to test the research hypotheses.

**Results:**

In total, 7626 HCWs participated in the present study. Accordingly, 73.2 and 26.8% of the workers were female and male, respectively. As well, 57.8% of the respondents were nurses and 14.4% of the cases were clinicians. Moreover, 44.8% of the participants had thus far worked in isolation wards and 40.3% of these individuals reported working for 4–8 hours with COVID-19 patients. The prevalence rate of OB was 18.3%. Besides, 34.2, 48.7, and 56.1% of the respondents had severe levels of emotional exhaustion (EE), higher depersonalization (DP), and decreased sense of personal accomplishment (PA), respectively. Besides, the HCWs at the age range of 20 to 30, having female gender, no children, and a bachelor’s degree, and working in isolation wards showed the higher levels of OB with reference to the Chi-square test results (*p* < 0.001). Accordingly, the statistical test outcomes demonstrated that a history of physical illnesses (*p* = 0.001) and psychiatric disorders (*p* = 0.044) could be the best predictor of OB throughout the first peak of the COVID-19 pandemic.

**Conclusion:**

Regarding the high prevalence rate of OB among the HCWs and the remaining COVID-19 journey in Iran, health care managers are recommended to orient the required management and coping strategies toward improving mental health in these individuals.

## Background

The novel coronavirus disease 2019 (COVID-19) [1], caused by severe acute respiratory syndrome (SARS)-associated coronavirus, was firstly reported in Wuhan, China, in December 2019, and quickly spread around the world, leading to global health threats and emergencies [2, 3]. Based on the World Health Organization (WHO) statistics on March 10, 2022, so far 7,107,167 cases with COVID-19 confirmed have been detected in Iran, with 138,433 deaths, within approximately 2 years from the onset of the COVID-19 pandemic [1].

In line with a Spanish study, 4.5% of HCWs had suffered from acute stress during the COVID-19 outbreak [4]. Currently, all health care systems are facing some challenging conditions, including numerous physical-psychosocial stressors, and even conflicts in professional ethics, sense of responsibility, and fear of being infected and transferring it to their own families, no childcare support, shortages of personal protective equipment, the rapid spread of the disease, failures in most nations to meet demands, and insufficient time to cope with this situation [3, 5–8]. A systematic review, reflecting on COVID-19 and its mortality rate in HCWs, in April 2020, had further established that the number of infected workers ranged from 1.03 to 10.7%, according to the statistics reported in each country. On the other hand, regarding the death rates, there were up to 605 HCWs all over the world, who had already lost their lives at the time of the pandemic [9]. Another systematic review and meta-analysis of the articles published in 2020 had similarly validated that the prevalence rate of the infection among HCWs was 11 and 7% with reference to the reverse transcription-polymerase chain reaction (RT-PCR) and the antibody screening test results, respectively [10]. Furthermore, one survey had revealed that thousands of workers infected with COVID-19 had expired worldwide [11].

As defined by the WHO in the 11th revision of the International Classification of Diseases (ICD-11), occupational burnout (OB) is “a syndrome resulting from chronic workplace stress that has not been successfully managed” [12–14]. This phenomenon also represents not only the manifestations of negative emotions but also the absence of positive ones [3]. OB is further characterized by three features, i.e., severe levels of emotional exhaustion (EE), higher depersonalization (DP), and decreased sense of personal accomplishment (PA) [15]. A systematic review in this domain had thus confirmed the significant development of OB among HCWs during the SARS and Middle East respiratory syndrome (MERS) epidemics [16]. Working in isolation wards, having special protective clothing on, higher exposure to COVID-19 patients, increased levels of workload during 1 month or long shifts, and fear of being infected and transferring it to their own families could accordingly have considerable negative consequences on mental health in HCWs, augmenting the possibility of being susceptible to OB [2, 13, 17, 18].

The results of one study had similarly revealed that 81.6% of the nurses had undergone mild OB, and 43.5–62.0% of the cases had been subjected to moderate-to-severe OB during COVID-19 [3]. Nevertheless, a systematic review had reported the prevalence rate of OB by 28% among the frontline [19], and the severity of this condition could be significantly associated with various individual (personality and self-esteem), social (lack of support and nationality), and organizational (job stress and workload) factors [20].

Of note, OB is assumed as a major global health problem, resulting in decreased mental health and well-being in HCWs as well as lower motivation to provide health care services, reduced quality of care delivered to patients, higher medical errors, and an elevated rate of absenteeism and turnover at the time of the pandemic [5, 18, 21]. Besides, HCWs with OB are more likely to feel dissatisfied with their positions and even look for other ones [21]. Highlighted in the related literature, hospital wards have been regarded as the most effective factor in OB severity [22, 23], so that, among the HCWs, nurses working in emergency wards and intensive care units (ICUs) have been more supposed to suffer from OB due to the higher levels of stress, workload, and exposure to patients with COVID-19 confirmed [24].

Based on the results of a survey in China, some factors, such as personnel agency, working experience for 5 years or lower, three or more night shifts weekly, working in isolation wards, and having contacts with medical staff confirmed or suspected around, were among the statistically significant predictors of OB in nurses [2]. Correspondingly, another study had found that OB could be correlated with nurses’ resilience, parenting stress, marital status, number of children, employment type, and gender, so that these individuals had reported higher levels of OB, less resilience, and higher tensions in terms of relationships with their children [25].

Depression, anxiety, and stress are additionally among the important psychological problems that may even give rise to emotional disturbance as the predisposing factor of OB [25, 26]. In this regard, there is a positive significant correlation between OB and anxiety and depression, according to the results of some studies, so that 77.1% of OB could be related to anxiety or depression, and 84.0% of anxiety and depression was associated with OB [27]. The results of one survey in Iran had further indicated that employment status, experience in caring for infected patients, hospital resources, and workplace stress were among the considerably significant risk factors of OB during the COVID-19 pandemic [28].

Even if a wide range of studies have been thus far fulfilled regarding OB all over the world and in Iran, as highlighted in the literature, working environments are completely different in each setting over the course of the pandemic as the most important factor shaping HCWs’ functions. As stated in the survey by Guan, OB could be significantly associated with the working environment perceptions [29]. Some limitations mentioned in the related literature in Iran have been also small sample size, no sample selection from other regions of the country [28], as well as limited socio-demographic and work-related data assessments [30]. In addition, no study has so far assessed OB among HCWs in all provinces of Iran, to the best of the authors’ knowledge. Therefore, the study results had implications and high generalization value as an effective guideline for health care managers. Accordingly, this study aimed to investigate the prevalence rate of OB in Iranian HCWs at the time of the COVID-19 pandemic.

## Materials and methods

### Study design and participants

In this web-based cross-sectional survey, a total number of 7626 HCWs in 31 provinces of Iran was recruited through the convenience sampling method, from April 6 to May 30, 2020. All the workers agreed to participate in the study.

### Instruments and data collection

A web-based questionnaire in Persian was completed using an online platform, developed by Sadra Rayaneh Novin Tabarestan Engineering Co., Mazandaran, Iran. For data collection purposes, the questionnaire was distributed via cyberspace, including popular messaging apps, i.e., WhatsApp, Instagram, and Short Message Service (SMS). In this study, two main instruments were accordingly applied, viz. a socio-demographic information form and the Maslach Burnout Inventory (MBI). Finally, the respondents’ scores were automatically calculated and promptly reported, as the questionnaires were received. Furthermore, the HCWs, obtaining high scores on the MBI (namely, unfavorable mental health), were recommended to contact the 4030 Call System, designed by Iran’s Ministry of Health and Medical Education, for free-of-charge consultations and read two stress management booklets available in this regard.

### Socio-demographic information form

The socio-demographic information form contained items, such as gender, age, marital status, place of living, level of education, academic major, working position, years of working experience, a history of mental and physical problems, number of children, and hospital wards (namely, inpatient or outpatient ones). As well, some items about COVID-19 were investigated in this study.

### MBI

The MBI was a 22-item questionnaire, as a standard tool to measure burnout, including OB [3, 31]. This scale was comprised of three dimensions, viz. EE, DP, and PA [32] scored on a seven-point Likert-type scale, ranging from 0 to 6. In this sense, score 0 meant “never occurs to me” and score 6 denoted “occurs to me every day”. The EE dimension was also made up of nine items (no. 1, 2, 3, 6, 8, 13, 14, 16, and 20), whose total score ranged from 0 to 54. The scores 0–18 also showed low OB, 19–26 represented moderate OB, and the values above 26 referred to high OB. Likewise, the DP dimension consisted of five items (no. 5, 10, 11, 15, and 22) with a total score ranging from 0 to 30, so that the scores 6–9 indicated moderate OB, and those higher than 9 pointed to high OB. The PA dimension was additionally comprised of eight items (no. 4, 7, 9, 12, 17, 18, 19, and 21), whose overall score was from 0 to 48 so that the scores 34–39 denoted moderate OB, and the values lower than 34 showed high OB. According to the MBI total score, burnout was divided into no OB (< 50), mild OB (50–75), moderate OB (76–100), and severe OB (> 100) [3]. As well, the high scores of the EE and DP dimensions and the low values of the PA one referred to OB [33, 34]. The internal consistency coefficients of the OB sub-scales had been further reported by α = 0.85, α = 0.82, and α = 0.52 for EE, DP, and PA, respectively [31]. The reliability coefficient of the questionnaire had been already established for the first time in Iran, using the test-retest method, and its reliability had been measured by Cronbach’s alpha coefficient of 0.78 and 0.86, respectively [35]. In the present study, the reliability had been confirmed by Cronbach’s alpha coefficients of α = 0.88, α = 0.81, α = 0.61, and α = 0.79 for the EE, PA, DP, and OB dimensions, respectively.

Upon receiving the ethics committee approval, the questionnaire was distributed. First, ethical aspects, including the codes of ethics and the privacy of responses, were clarified. Then, the OB scores were displayed, and the relevant data, such as management strategies to deal with OB and the corresponding author’s e-mail were provided in this regard.

### Statistical analysis

The data analysis was performed using the Statistical Package for the Social Sciences (SPSS, ver. 24) software (SPSS Inc., IBM Corporation, USA). To identify the missing data, they were also checked and cleaned before further analysis. Descriptive analyses, mean ± standard deviation (SD), were also utilized to illustrate the quantitative variables. Moreover, frequency and percentage were applied to describe the qualitative ones. The prevalence rate of OB caused by COVID-19 was additionally reported. The univariate and multivariate logistic regression models were correspondingly exploited to investigate the potential predictors of OB during the COVID-19 pandemic. As well, p<0.05 was considered statistically significant.

## Results

The HCWs’ socio-demographic characteristics are shown in Table 1. In total, 7626 HCWs participated in the present study. In this respect, there were 1098 (14.4%) clinicians, 4409 (57.8%) nurses, and 2119 (27.8%) HCWs involved in other fields. The majority of the HCWs were female (73.2%), married (55.8%), working as a nurse (57.8%), and aged 20–30 (56.2). Moreover, 8.4 and 19.6% of the respondents reported a history of psychiatric disorders and physical illnesses, respectively. In addition, 44.8% of the HCWs were working in isolation wards for COVID-19 and 40.3% of them were working for 4–8 hours a day with the patients confirmed.

**Table 1:** Sociodemographic characteristics of participants

Variables		Frequency (percent), ***N*** = 7626
**Age (Years)**	20–30	4287 (56.2)
	31–40	2286 (30.0)
	41–50	829 (10.8)
	> 50	224 (3.0)
**Gender**	Male	2044 (26.8)
	Female	5582 (73.2)
**Marital status**	Single	3176 (41.6)
	Married	4257 (55.8)
	Divorced or widowed	193 (2.5)
**Having children**	Yes	2694 (35.3)
	No	4932 (64.7)
**Level of education**	Undergraduate	888 (11.6)
	Bachelor’s degree	5323 (69.8)
	Master’s and Ph.D. degree	628 (8.3)
	General and special professional doctorate	787 (10.3)
**Occupation**	Clinician	1098 (14.4)
	Nurse	4409 (57.8)
	Others	2119 (27.8)
**Working in isolated units**	Yes	3420 (44.8)
	No	4206 (55.2)
**Working hours with COVID-19 patients**	< 4	3196 (41.9)
	4–8	3074 (40.3)
	> 8	1356 (17.8)
**History of physical illness**	Yes	1497 (19.6)
	No	6129 (80.4)
**History of psychiatric disorders**	Yes	639 (8.4)
	No	6987 (91.6)

The prevalence rate of OB was also 18.3% in the HCWs over the course of the COVID-19 pandemic. Besides, most of the HCWs had experienced severe OB in all three dimensions. Therefore, 34.2, 48.7, and 56.1% of the participants reported severe EE, DP, and PA, respectively (Table 2).

**Table 2:** The prevalence of occupational burnout among HCWs

Severity	Mild	Moderate	Severe
Variable			
Emotional exhaustion	2604 (34.1%)	2410 (31.7%)	2605 (34.2%)
Depersonalization	716 (9.4%)	3192 (41.9%)	3711 (48.7%)
Personal accomplishment	1900 (24.9%)	1449 (19.0%)	4270 (56.1%)
Yes			No
Having occupational burnout		1395 (18.3%)	6231 (81.7%)

The HCWs’ OB during the COVID-19 pandemic, stratified by the socio-demographic variables, is outlined in Table 3. The Chi-square test results also showed that OB was significantly different in terms of some variables, such as age, gender, having children, level of education, and working in isolation wards (*p* < 0.001). Accordingly, female participants, having no children, at the age range of 20 to 30, holding a bachelor’s degree, and working as a nurse in isolation wards had higher levels of OB as compared with others.

**Table 3:** Occupational burnout in participants during COVID-19 outbreak among HCWs stratified by socio-demographic variables

Variables		Emotional Exhaustion				Depersonalization				Personal Accomplishment				Occupational Burn out		
		Mild N (%)	Moderate N (%)	Severe N (%)	*p*-value*	Mild N (%)	Moderate N (%)	Severe N (%)	*p*-value*	Mild N (%)	Moderate N (%)	Severe N (%)	*p*-value*	Yes	No	*p*-value*
Gender	Female	1727 (22.6%)	1802 (23.6%)	2053 (26.9%)	< 0.001	499 (6.5%)	2388 (31.3%)	2695 (35.3%)	0.011	1315 (17.2%)	1088 (14.3%)	3179 (41.7%)	< 0.001	1090 (14.3%)	4492 (58.9%)	< 0.001
	Male	881 (11.6%)	609 (8.0%)	554 (7.3%)		217 (2.8%)	808 (10.6%)	1019 (13.4%)		587 (7.7%)	362 (4.7%)	1095 (14.4%)		305 (4.0%)	1739 (22.8%)	
Having children	Yes	1045 (13.7%)	851 (11.2%)	798 (10.5%)	0 < 0.01	242 (3.2%)	1365 (17.9%)	1087 (14.3%)	< 0.001	872 (11.4%)	558 (7.3%)	1264 (16.6%)	< 0.001	352 (4.6%)	2342 (30.7%)	< 0.001
	No	1563 (20.5%)	1560 (20.5%)	1809 (23.7%)		474 (6.2%)	1831 (24.0%)	2627 (34.4%)		1030 (13.5%)	892 (11.7%)	3010 (39.5%)		1043 (13.7%)	3889 (51.0%)	
Age	20–30	1322 (17.3%)	1366 (17.9%)	1599 (21.0%)	< 0.001	425 (5.6%)	1449 (19.0%)	2413 (31.6%)	< 0.001	822 (10.8%)	804 (10.5%)	2661 (34.9%)	< 0.001	935 (12.3%)	3352 (44.0%)	< 0.001
	31–40	817 (10.7%)	731 (9.6%0	738 (9.7%)		211 (2.8%)	1107 (14.5%)	968 (12.7%)		617 (8.1%)	436 (5.7%)	1233 (16.2%)		364 (4.8%)	1922 (25.2%)	
	41–50	356 (4.7%)	246 (3.2%)	227 (3.0%)		64 (0.8%)	501 (6.6%)	264 (3.5%)		347 (4.6%)	169 (2.2%)	313 (4.1%)		80 (1.0%)	749 (9.8%)	
														16 (0.2%)	208 (2.7%)	
	> 50	113 (1.5%)	68 (0.9%)	43 (0.6%)		16 (0.2%)	139 (1.8%)	69 (0.9%)		116 (1.5%)	41 (0.5%)	67 (0.9%)				
Marital status	Single	1038 (13.6%)	978 (12.8%)	1160 (15.2%)	0.001	284 (3.7%)	1170 (15.3%)	1722 (22.6%)	< 0.001	671 (8.8%)	571 (7.5%)	1934 (25.4%)	< 0.001	682 (8.9%)	2494 (32.7%)	< 0.001
	Married	1503 (19.7%)	1382 (18.1%)	1372 (18.0%)		414 (5.4%)	1944 (25.5%)	1899 (24.9%)		1174 (15.4%)	844 (11.1%)	2239 (29.4%)		670 (8.8%)	3587 (47.0%)	
	Divorced/widowed	67 (0.9%)	51 (0.7%)	75 (1.0%)		18 (0.2%)	82 (1.1%)	93 (1.2%)		57 (0.7%)	35 (0.5%)	101 (1.3%)		43 (0.6%)	150 (2.0%)	
Work experience	0–5	1267 (16.6%)	1237 (16.2%)	1405 (18.4%)	< 0.001	403 (5.3%)	1382 (18.1%)	2124 (27.9%)	< 0.001	780 10.2%)	734 (9.6%)	2395 (31.4%)	< 0.001	813 (10.7%)	3096 (40.6%)	< 0.001
	6–10	527 (6.9%)	492 (6.5%)	566 (7.4%)		148 (1.9%)	664 (8.7%)	773 (10.1%)		377 (4.9%)	286 (3.8%)	922 (12.1%)		314 (4.1%)	1271 (16.7%)	
	11–15	376 (4.9%)	372 (4.9%)	352 (4.6%)		87 (1.1%)	535 (7.0%)	478 (6.3%)		318 (4.2%)	277 (3.0%)	555 (7.3%)		161 (2.1%)	939 (12.3%)	
	16–20	210 (2.8%)	171 (2.2%)	150 (2.0%)		44 (0.6%)	303 (4.0%)	184 (2.4%)		196 (2.6%)	104 (1.4%)	231 (3.0%)		65 (0.9%)	466 (6.1%)	
	21–25	126 (1.7%)	94 (1.2%)	90 (1.2%)		18 (0.2%)	190 (2.5%)	102 (1.3%)		132 (1.7%)	69 (0.9%)	109 (1.4%)		25 (0.3%)	285 (3.7%)	
	26–30	102 (1.3%)	45 (0.6%)	44 (0.6%)		16 (0.2%)	122 (1.6%)	53 (0.7%)		99 (1.3%)	30 (0.4%)	62 (0.8%)		17 (0.2%)	174 (2.3%)	
Level of education	Undergraduate	412 (5.4%)	253 (3.3%)	223 (2.9%)	< 0.001	117 (1.5%)	379 (5.0%)	392 (5.1%)	< 0.001	260 (3.4%)	171 (2.2%)	457 (6.0%)	< 0.001	99 (1.3%)	789 (10.3%)	< 0.001
	Bachelor’s degree	1677 (22.0%)	1738 (22.8%)	1908 (25.0%)		494 (6.5%)	2117 (27.8%)	2712 (35.6%)		1196 (15.7%)	1014 (13.3%)	3113 (40.8%)		1058 (13.9%)	4265 (55.9%)	
	Master’s and Ph.D. degree	240 (3.1%)	202 (2.6%)	186 (2.4%)		58 (0.8%)	320 (4.2%)	250 (3.3%)		197 (2.6%)	105 (1.4%)	326 (4.3%)		86 (1.1%)	542 (7.1%)	
	General & special professional doctorate	279 (3.7%)	218 (2.9%)	290 (3.8%)		47 (0.6%)	380 (5.0%)	360 (4.7%)		249 (3.3%)	160 (2.1%)	378 (5.0%)		152 (2.0%)	635 (8.3%)	
Occupation	Clinician	406 (5.3%)	312 (4.1%)	380 (5.0%)	< 0.001	95 (1.2%)	497 (6.5%)	506 (6.6%)	< 0.001	322 (4.2%)	213 (2.8%)	563 (7.4%)	< 0.001	199 (2.6%)	899 (11.8%)	< 0.001
	Nurse	1323 (17.3%)	1476 (19.4%)	1610 (21.1%)		374 (4.9%)	1645 (21.6%)	2390 (31.3%)		970 (12.7%)	830 (10.9%)	2609 (34.2%)		904 (11.9%)	3505 (46.0%)	
	Others	879 (11.5%)	623 (8.2%)	617 (8.1%)		247 (3.3%)	1054 (13.8%)	818 (10.1%)		610 (8.0%)	407 (5.3%)	1102 (14.5%)		292 (3.8%)	1827 (23.9%)	
Working in isolated wards	Yes	1026 (13.5%)	1082 (14.2%)	1312 (17.2%)	< 0.001	262 (3.4%)	1301 (17.1%)	1857 (24.4%)	< 0.001	850 (11.1%)	670 (8.8%)	1900 (24.9%)	0.504	724 (9.5%)	2696 (35.4%)	< 0.001
	No	1582 (20.7%)	1329 (17.4%)	1295 (17.0%)		454 (6.0%)	1895 (24.8%)	1857 (24.4%)		1052 (13.8%)	780 (10.2%)	2374 (56.4%)		671 (8.8%)	3535 (46.4%)	
Working hours with COVID-19 patients	< 4	1326 (17.4%)	995 (13.1%)	875 (11.5%)	< 0.001	374 (4.9%)	1446 (18.9%)	1376 (18.1%)	< 0.001	771 (10.2%)	576 (7.6%)	1849 (24.2%)	0.049	452 (6.0%)	2744 (35.9%)	< 0.001
	4–8	881 (11.6%)	1024 (13.5%)	11.69 (15.3%)		225 (2.9%)	1264 (16.6%)	1585 (20.7%)		776 (10.2%)	612 (8.1%)	1686 (22.1%)		626 (8.2%)	2448 (11.6%)	
	> 8	401 (5.3%)	392 (5.1%)	563 (7.4%)		117 (1.5%)	486 (6.4%)	753 (9.9%)		355 (4.7%)	262 (3.4%)	739 (9.7%)		317 (4.2%)	1039 (13.6%)	
History of physical illness	Yes	429 (5.6%)	467 (6.1%)	604 (7.9%)	< 0.001	124 (1.6%)	629 (8.2%)	744 (9.8%)	0.245	421 (5.5%)	282 (3.7%)	794 (10.4%)	0.005	295 (3.9%)	1202 (15.8%)	0.117
	No	2179 (28.6%)	1947 (25.5%)	2003 (26.3%)		592 (7.8%)	2567 (33.7%)	2970 (38.9%)		1481 (19.4%)	1168 (15.3%)	3480 (45.6%)		1100 (14.4%)	5029 (65.9%)	
History of psychiatric disorders	Yes	139 (1.8%)	177 (2.3%)	323 (4.2%)	< 0.001	52 (0.7%)	236 (3.1%)	351 (4.6%)	0.004	102 (1.3%)	114 (1.5%)	423 (5.5%)	< 0.001	181 (2.4%)	458 (6.0%)	< 0.001
	No	2469 (32.4%)	2234 (29.3%)	2284 (30.0%)		664 (8.7%)	2960 (38.8%)	3363 (44.1%)		1800 (23.6%)	1336 (17.5%)	3851 (50.5%)		1214 (15.9%)	5773 (75.7%)	

*Chi square test

Statistical analysis showed most of the female HCWs experienced severe EE (26.9%), DP (35.3%), and PA (41.7%). However, most of the HCWs who had no children had a severe levels of EE (23.7%), DP (34.4%) and, PA (39.5%). Most of the 20 to 30-years-old HCWs had severe EE (21.0%), DP (31.6%) and, PA (34.9%). 25.0, 35.6, and 40.8% of HCWs with bachelor’s degrees experienced the severe level of EE, DP, and PA, respectively. Also, nurses experienced the severe levels of EE, DP, and PA in comparison to other HCWs.

In view of that, logistic regression analysis was used to describe the OB predictors (Table 4). The statistical test results additionally demonstrated a history of physical illnesses (odds ratio [OR]: 0.440, 95% confidence interval [CI]: 0.270–0.718, *p* = 0.001) and psychiatric disorders (OR: 0.537, 95% CI: 0.293–0.985, *p* = 0.044), as the predictors of OB during the first peak of COVID-19. The logistic regression model outcomes similarly revealed a non-significant difference between clinicians and nurses (OR: 0.756, 95% CI: 0.373–1.530, *p* = 0.437 vs. OR: 1.745, 95% CI: 0.456–6.684, *p* = 0.416, respectively) and having children and others (OR: 1.250, 95% CI: 0.686–2.279, *p* = 0.466) in terms of OB.

**Table 4:** Logistic regression for predictors of occupational burnout

Variables		Univariate			Multivariate		
		OR	95% CI	*P*-value	OR	95% CI	*P*-value
Gender	Male	Ref.	Ref.	Ref.	Ref.	Ref.	Ref.
	Female	0.770	0.683–0.867	< 0.001	1.177	0.746–1.856	0.484
Age	20–30	0.626	0.447–0.876	0.006	0.804	0.340–1.901	0.619
	31–40	0.768	0.545–1.083	0.133	0.685	0.322–1.457	0.326
	41–50	0.833	0.577–1.204	0.331	1.245	0.600–2.585	0.556
	> 50	Ref.	Ref.	Ref.	Ref.	Ref.	Ref.
Marital status	Single	1.096	0.795–1.510	0.576	1.007	0.307–3.309	0.991
	Married	1.219	0.887–1.676	0.223	1.158	0.373–3.590	0.800
	Divorced/widowed	Ref.	Ref.	Ref.	Ref.	Ref.	Ref.
Having children	Yes	1.215	1.090–1.355	< 0.001	1.250	0.686–2.279	0.466
	No	Ref.	Ref.	Ref.	Ref.	Ref.	Ref.
Level of education	Undergraduate	1.533	1.148–2.047	0.004	0.316	0.016–6.189	0.448
	Bachelor’s degree	1.105	0.937–1.304	0.234	1.088	0.330–3.591	0.889
	Master’s and Ph.D. degree	1.877	0.900–1.304	0.093	1.469	0.263–8.195	0.661
	General & special professional doctorate	Ref.	Ref.	Ref.	Ref.	Ref.	Ref.
Occupation	Clinician	1.321	0.775–2.252	0.306	0.756	0.373–1.530	0.437
	Nurse	2.209	0.632–7.716	0.214	1.745	0.456–6.684	0.416
	Other	Ref.	Ref.	Ref.	Ref.	Ref.	Ref.
Working hours with COVID-19 patients	< 4	1.982	1.718–2.285	< 0.001	1.163	0.592–2.284	0.661
	4–8	1.196	1.043–1.372	0.011	0.793	0.416–1.510	0.480
	> 8	Ref.	Ref.	Ref.	Ref.	Ref.	Ref.
Working in isolated wards	Yes	0.743	0.670–0.823	< 0.001	0.988	0.596–1.638	0.962
	No	Ref.	Ref.	Ref.	Ref.	Ref.	Ref.
History of physical illness	Yes	0.653	0.577–0.738	< 0.001	0.440	0.270–0.718	0.001
	No	Ref.	Ref.	Ref.	Ref.	Ref.	Ref.
History of psychiatric disorder	Yes	0.621	0.523–0.737	< 0.001	0.537	0.293–0.985	0.044
	No	Ref.	Ref.	Ref.	Ref.	Ref.	Ref.

## Discussion

The main purpose of this study was to investigate the prevalence rate of OB in HCWs at the time of the COVID-19 pandemic. The study findings established that 18.3% of the HCWs had experienced OB. In addition, 34.2, 48.7, and 56.1% of these workers presented severe levels of EE, DP, and PA, respectively. Like the present study, these frequencies were equal to 40.7, 30.2, and 36.4% in an Italian survey during the first 4 months of the COVID-19 pandemic, respectively [36]. Moreover, these values had been reported respectively by 38.5, 31.2, and 33.6% in one study in Saudi Arabia, 10 months after the onset of COVID-19 [37]. The prevalence rate of OB was also slightly different in the study by Jalili et al. in Iran [30]. Accordingly, they had reported the prevalence rate of OB and the high levels of EE, DP, and PA by 53.0, 50.1, 13.2, and 85.5%, respectively. The different levels of OB in the given study were associated with the fact that the given phenomenon had been simply assessed during the first 2 months of COVID-19 just in one city in Iran and the response rate had been moderate. However, the present survey was conducted in all provinces of Iran with larger sample size. Overall, the discrepancy in the prevalence rates could be attributed to the pandemic time. One to 2 years after the SARS outbreak, Maunder et al. had also revealed that OB had elevated among HCWs [38], so the assessment time could be of utmost importance. On the other hand, OB during the pandemic conditions could be time-dependent. As COVID-19 had fluctuated in Iran, the HCWs might have experienced the peaks of the disease, and this might have affected their mental health. In addition, the controlled COVID-19 condition was not similar in different countries and even the cities in the same country. Therefore, comparisons sound complicated.

Besides, the study results confirmed that the nurses had been subjected to significantly higher levels of EE, DP, and PA than other HCWs during the pandemic. In the survey by Naldi et al. during the COVID-19 outbreak, the statistical test results had revealed no significant difference between the nurses and other HCWs although the nurses had suffered from more severe distress than others [36]. In one other study in Iran, medical residents and then nurses had significantly undergone higher levels of EE and PA all through the first peak of COVID-19 [30]. Occupational stress could be thus one of the predictors of OB and nurses could endure stress and other mental pressures, especially during the COVID-19 pandemic [39], such as conflicts with supervisors, patients, and their families, role overload, and increased working hours [40, 41].

However, Barello et al. had reported that nurses had faced higher prevalence rates of OB compared with other workers [42]. This discrepancy might be related to the fact that the nurses working in different wards had not suffered from similar OB. For example, Ahmadi et al. had demonstrated that the ICU and emergency ward nurses were more likely to be influenced by OB than those working in orthopedic and hemodialysis ones [43]. Besides, one study had reported that surgical residents could frequently experience higher levels of OB than others [44], however, neurology residents had undergone the same condition in Kulik et al [45] Another survey in Iran had further established that residents working in emergency and surgical wards had been subjected to more OB as compared with others [46]. Even though the long working hours per week for the medical residents were expected, they were significantly correlated with OB in pediatric residents, as reported by Treluyer and Tourneux [47].

In the present study, OB was significantly different in terms of direct contact with COVID-19 patients and the hours spend with them by each worker. Most of the HCWs also reported that OB was not effective in isolation wards. But, direct contact with COVID-19 patients and long working hours were significantly associated with OB in Roslan et al [48] A study in Iran had also found that the OB scores had been higher in frontliners in comparison with non-frontline HCWs [28]. Although the workers involved in isolation wards were using special protective clothing and equipment, anxiety had merely occurred due to no awareness of definite COVID-19 in the non-isolation wards. In Mira et al., the acute stress score had been higher in hospitals with higher COVID-19 death rates [4]. In the study by Koutsimani et al., a significant relationship had been further observed between anxiety and OB [49]. Guixia and Hui had similarly shown that burnout was positively correlated with anxiety during this pandemic [3]. Correspondingly, Azizi et al. had reported that working hours spent with COVID-19 patients were significantly associated with anxiety induced by COVID-19 [50].

One of the variables that had not been assessed in previous studies in Iran was the working experience with regard to OB during COVID-19. In the present study, the working experience was also significantly different among the HCWs in terms of OB. Therefore, the lower the working experience, the greater the OB. Nevertheless, one other survey in Iran had demonstrated that working experience was not significantly correlated with the OB severity [14]. The lack of similar studies also made the comparisons of the results in this survey difficult.

Besides, the statistical results of the present study revealed a significant discrepancy between the male and female participants regarding OB. In this sense, the female HCWs experienced more OB than males. These results were in line with the reports by Alanazi et al [37] In the survey by Alrawashdeh et al., female clinicians had also faced higher OB during the COVID-19 pandemic [51]. High occupational demand might have been related to high OB. It seems that reducing occupational demands is a protective factor among females. As a result, they may be subjected to higher levels of OB over the course of COVID-19 [52]. Likewise, married HCWs had experienced significantly high levels of OB while it was not so in other research [15, 30]. As married people had to play different roles, including responsibilities at home and childcare, they might have been subjected to higher burnout [53]. In the present study, having no children was significantly correlated with a higher prevalence rate of OB, in agreement with the survey by Jalili et al. [30], but contrasting the reports by Huang et al. and Lai et al [54, 55] Childless people might also live through more depression and mental health problems [56]. Moreover, they may not receive appropriate social support [57] and be more vulnerable to burnout.

The sample size in the present study was larger compared with that in other surveys, which boosted the generalizability of the results to manage the COVID-19 pandemic. Regarding the high prevalence rate of OB among the HCWs and the unfinished COVID-19 conditions in Iran, health care managers are recommended to orient the management strategies toward improving mental health in such workers [58]. As regards, Iran faces multiple peaks of this infection, so it is suggested to assess mental health, particularly OB, at different times of the pandemic. Health care managers should also adopt multiple coping strategies, including spiritual health promotion, to tackle OB [59–61], and use digital tools to provide emotional support for controlling acute stress and emotional distress during and after the COVID-19 pandemic [62]. One limitation of the present study was the application of social media to collect the data, so not everyone might have access to such media or have much time to tap them.

## Conclusion

The prevalence rate of OB was 18.3% in the Iranian HCWs over the course of the COVID-19 pandemic. Special attention, including screening and organizational support, should thus target the cases with less experience, female gender, having no children, aged 20–30, holding a bachelor’s degree, and working as a nurse in isolation wards of the hospital, who might have higher levels of OB in relation to others.

## Data Availability

The datasets used and/or analyzed during the present study are available from the corresponding author on reasonable request.

## Abbreviations

COVID-19: Coronavirus disease 2019
HCWs: Health care workers
OB: Occupational burnout
MBI: Maslach Burnout Inventory
EE: Emotional exhaustion
DP: Depersonalization
PA: Personal accomplishment
SARS: Severe acute respiratory syndrome
MERS: Middle East respiratory syndrome
WHO: World Health Organization
RT-PCR: Reverse transcription-polymerase chain reaction
ICD-11: 11th revision of the International Classification of Diseases
ICU: Intensive care unit
SMS: Short message service
SPSS: Statistical package for the social sciences
OR: Odds ratio
CI: Confidence interval
